# Different clinical phenotypes of AQP4-IgG positive NMOSD in two first degree relatives

**DOI:** 10.1186/s12883-023-03366-5

**Published:** 2023-09-04

**Authors:** Salvatore Ssemmanda, Abdu Kisekka Musubire

**Affiliations:** 1 C-Care International Hospital Kampala, Plot 4686 Barnabas Road, Kampala, Uganda; 2 Aga Khan University Hospital, Kampala Medical Centre, Kampala, Uganda; 3 Kiruddu National Referral Hospital, Kampala, Uganda

**Keywords:** Neuromyelitis optica spectrum disorder, Familial NMOSD, NMOSD genetics, NMOSD sub-saharan Africa, Familial NMOSD sub-saharan Africa.

## Abstract

**Background:**

Neuromyelitis optica spectrum disorder (NMOSD) are autoimmune inflammatory disorders of the CNS in which patients have severe relapses of optic neuritis and myelitis. Aquaporin-4 antibody (AQP4-IgG) positive NMOSD has not been reported in members of the same family in Sub Saharan Africa. We report the uncommon scenario in which both a Ugandan HIV positive woman and her HIV negative daughter were diagnosed with AQP4-IgG positive NMOSD. We discuss pathogenic mechanisms that may underlie familial presentation of AQP4-IgG positive NMOSD.

**Case Presentation:**

Case 1, a 54-year-old female teacher with a 20-year history of HIV infection and virally suppressed on Tenofovir, Lamivudine and Dolutegravir HAART regimen, presented with 8 months of progressive quadriparesis and urinary incontinence with a T6 sensory level. She had gadolinium enhancing longitudinally extensive transverse myelitis on MRI and was AQP4-IgG positive on serum studies. She received IV Methylprednisone 1 g daily for 3 days as a pulse and was continued on tapering doses of oral Prednisone with maintenance doses of Azathioprine. She showed slow improvements in limb motor function. Her daughter, case 2, is a 35-year-old HIV negative nutritionist, independently ambulant, with no known comorbidities or precedent autoimmune disease. She presented 1 year before her mother’s AQP4-IgG positive NMOSD diagnosis with 7 months history of bilateral visual loss of rapid onset, with gadolinium-enhancing optic nerves on Brain and orbit MRI, in keeping with bilateral optic neuritis. She was AQP4-IgG positive on serum studies. She stabilized on tapered doses of oral Prednisone and continues daily oral Azathioprine with moderate improvement in her vision and no further relapses as yet.

**Conclusions:**

We add to existing literature and hypothesize that NMOSD appears to show a complex genetic background. To our knowledge, this is the first report in Sub-Saharan Africa, of familial AQP4-IgG positive NMOSD presenting with clinical heterogeneity between first degree relatives. A better understanding of the pathogenic mechanisms involved, including genome wide studies for particular risk loci for familial NMOSD, will be pivotal for future preventative and therapeutic strategies.

## Background

Neuromyelitis optica spectrum disorder (NMOSD) are autoimmune inflammatory disorders of the CNS in which patients have severe relapses of optic neuritis and myelitis. Familial occurrence of NMOSD is estimated at 3%. Aquaporin-4 (AQP4) is a serum IgG autoantibody marker which with suggestive clinical diagnostic criteria, allows sensitive and specific diagnosis of NMOSD [1]. Aquaporin-4 antibody (AQP4-IgG)–positive NMOSD has not been reported in members of the same family in Sub Saharan Africa. We report the uncommon scenario in which a Ugandan middle aged HIV positive woman was diagnosed with AQP4-IgG positive NMOSD presenting with severe longitudinally extensive transverse myelitis one year after her HIV negative daughter had been diagnosed with AQP4-IgG positive NMOSD presenting with asymmetric bilateral moderately severe optic neuritis. We discuss pathogenic mechanisms that may underlie familial presentation of AQP4-IgG positive NMOSD.

## Case Presentation

### Case 1: mother

We evaluated a 54-year-old female teacher with a 20-year history of HIV infection, who was virally suppressed on Tenofovir, Lamivudine and Dolutegravir-HAART regimen. She had no precedent autoimmune disease and was constitutionally well when she developed progressively worsening severe mid upper back pain. Over the subsequent month, her symptoms were compounded by spasmic aches in her 4th and 5th left fingers, weakening grip in both hands, left proximal lower limb weakness, abnormal sensation to cold in her left hemibody and constipation. Within 4 months of symptom onset, she was using a wheelchair for assisted mobility. She was taking 60 mg of daily oral Prednisone for presumptive diagnoses of spinal cord ischemia, neurosarcoidosis or multiple sclerosis when she got briefly lost to follow up and got treatment for a left leg deep venous thrombosis.

She contacted S.S for a second opinion 8 months after initial symptom onset, reporting 6 weeks of insidious onset of urine incontinence and worsening paraparesis. She had normal sensorium, no meningism or cranial nerve deficits. Her fingers were flexed bilaterally with mild bilateral thenar and hypothenar atrophy. Her feet were in spastic plantar flexion. She was able to lift upper limbs against gravity but not against resistance and only moved her lower limbs on elimination of gravity. She had no clonus but had asymmetrically brisk lower limb reflexes, more in her right extremity, and a T6 sensory level. Her gait and coordination could not be fully tested.

Index whole spine MRI had shown longitudinally extensive T2-FLAIR intrinsic spinal cord hyperintensity with mild localized edema extending from C2 to T5 (Fig. A1). Repeat spinal imaging at 8 months revealed gadolinium enhancing spinal cord longitudinally extensive T2-FLAIR hyperintensity with localized edema extending from C2 to T4 (Fig. A2-A3). Brain imaging at both visits revealed only non-specific periventricular hyperintensities on T2-FLAIR MRI sequence (Fig. B). CSF analysis showed 0 neutrophils per microliter, 2 lymphocytes, 230 erythrocytes, normoglycorrhachia, protein of 39 mg/dL, negative oligoclonal bands. CSF was negative for HSV1/2, VZV, Enterovirus and HHV6 PCRs, EBV VL, TB GeneXpert, cryptococcal antigen, FTA-ABS and negative on microbial culture. CSF IgG index is not available in Uganda. Laboratory serologic testing revealed normal C-reactive protein (CRP) levels, positive antinuclear factor (titre, 1:160) and negative dsDNA antibody. Serum AQP4-IgG was positive (titre, 1:10). Myelin oligodendrocyte glycoprotein was not done. Chest X-ray was normal. A diagnosis of AQP4-IgG positive NMOSD was made.

Treatment with methylprednisolone 1 gram intravenously for 3 days resulted in slight improvement in proximal extremity strength in all four limbs. She continued oral Prednisone 60 mg once daily tapered by 5 mg monthly and oral Azathioprine up to 150 mg once daily. At one month follow-up, she was able to use her hands to eat, brush her teeth with difficulty and flex her knees in bed. She awaits the 3 month and 6 month follow ups.

**Fig.**
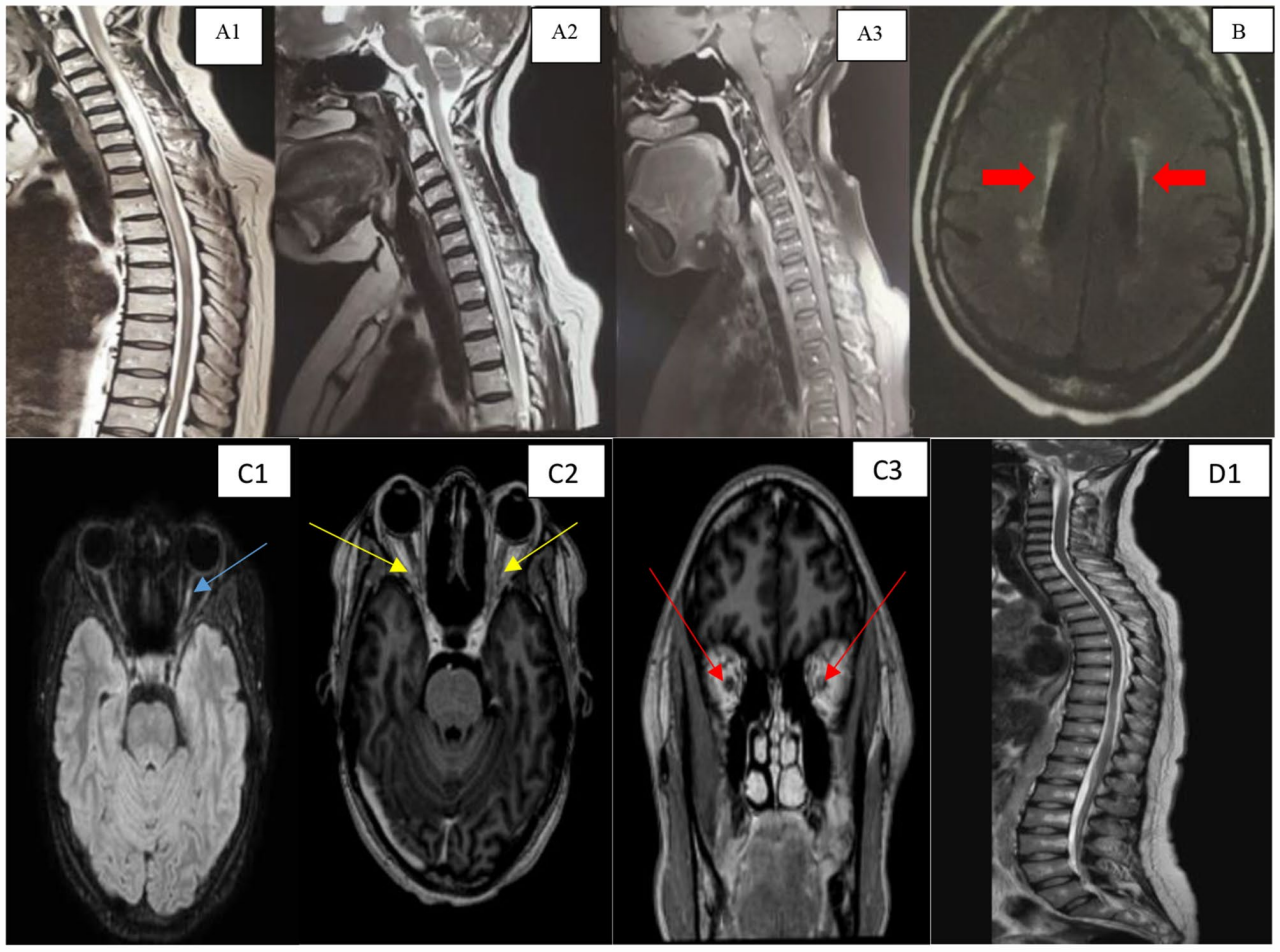
(**A1**) Sagittal T2-weighted MRI of case one’s cervical and thoracic spine demonstrating longitudinally extensive T2 hyperintensity from C2-T5 with localized spinal cord edema. (**A2**) Sagittal T2-weighted MRI of case one’s cervical and thoracic spine 8 months later, demonstrating longitudinally extensive T2 hyperintensity from C2-T4 with localized spinal cord edema. (**A3**) Sagittal T1 gadolinium enhanced MRI of case one’s cervical and thoracic spine at 8 months from initial symptom onset, demonstrating contrast enhancement in the cervical portions of her spinal cord. (**B**) Axial T2-FLAIR brain MRI of case one, showing periventricular hyperintensities (red arrows) with no significant other parenchymal abnormality. (**C1**), Axial T2-FLAIR optic nerve and brain MRI of case two demonstrating hyperintensity in the optic nerves, more evident in the left optic nerve (blue arrow). (**C2**), (**C3**) Axial and coronal T1-gadolinium enhanced optic nerve and brain MRIs of case two, demonstrating contrast enhancement in both optic nerves, more extensive in her left optic nerve (yellow and red arrows). (**D1**) Sagittal T2-weighted MRI of case two’s spinal cord, demonstrating normal spinal cord signal

### Case 2: daughter

Case’s daughter is a 35-year-old HIV-seronegative nutritionist, independently ambulant, with no known comorbidities or precedent autoimmune disease history. One year prior to her mother’s diagnosis of AQP4-IgG NMOSD, she suddenly developed a central scotoma in her right eye vision, three weeks after her first COVID vaccination dose of AstraZeneca and was started on 20 mg once daily oral Prednisone for right sided optic neuritis. She received her second dose of AstraZeneca 5 weeks later, about 3 weeks before developing sudden onset acute binocular blindness with painful eye movements.

Her first neurological consultation was 7 months after initial symptom onset. At the time, she also reported burning neuropathic pain of peripheral origin in both legs that was self-limited. She had normal sensorium, no meningism, and could only perceive light in both eyes. She had a right sided optic atrophy with a left sided swollen optic disc and no other cranial nerve deficits. The rest of her neurological exam was normal.

At disease onset, Brain CT scan and MRI studies were normal. Repeat MRI of the brain and optic nerves at symptom recurrence showed bilateral hyperintense optic nerves on T2-FLAIR (Fig. C1) and T1 gadolinium enhanced bilateral contrast enhancing optic nerves in their intra-orbital segments, left more than right (Fig. C2, C3). The rest of the brain was normal. MRI of her spinal cord (Fig. D1) was normal.

CSF analysis showed no pleocytosis, normoglycorrhachia, elevated protein of 100.8 mg/dL, negative Ziehl-Neelsen stain for acid fast bacilli, negative cryptococcal antigen and no microbial growth on CSF quantitative culture. Laboratory testing for systemic inflammatory markers (CRP and Erythrocyte Sedimentation Rate) was unremarkable. She was negative for antinuclear and extracted nuclear antigen antibodies. Serum AQP4-IgG was positive (Titre, > 1:32), myelin oligodendrocyte glycoprotein antibody was negative. Chest X-ray was normal. She was diagnosed with AQP4-IgG positive NMOSD.

She was initiated on oral Prednisone 60 mg daily, tapered off over 6 months, but continues oral Azathioprine 150 mg once daily. She has not had relapses or progression of her optic neuritis as yet and suggests that although her right eye vision has improved by only 10%, her left eye has improved by at least 70% to date. She is fully independent for all basic and instrumental activities of daily living.

## Discussion and conclusions

NMOSD are autoimmune inflammatory disorders of the CNS in which patients have severe relapses of optic neuritis and myelitis. Familial occurrence of NMOSD is estimated at 3% from a previous case series of 12 duplex families where 2 were of African ancestry and 4 had a mother and her child affected [1]. Additionally, anticipation phenomena are reported in familial NMOSD [2] as we also observed in our cases.

As described [1], our familial cases were clinically indistinguishable from sporadic cases but clinically presented differently, unlike some families described in earlier reports [1]. The factors responsible for this clinical heterogeneity, as sought in a study of 14 Asian mother-daughter pairs of familial NMOSD [2], are uncertain. We cannot directly ascribe case one’s more severe and delayed manifestation of NMOSD to HIV infection because although HIV associated NMOSD is described [3], HIV’s role in clinical heterogeneity of familial NMOSD is not. Furthermore, NMOSD onset and relapse post COVID-19 vaccination has been described [4] and therefore each one of our described cases may have an independent pathogenic mechanism for NMOSD but genetic factors are not excluded. Whether genetic predisposition to NMOSD has a role in the onset of the condition post COVID vaccination is also uncertain and warrants more studies.

Genetic predisposition to NMOSD is suggested by Human Leukocyte Antigen associations reported in Japanese populations (HLA-DR*03:01 and DPB1*0501) [1, 5] and French Caucasians [1] (HLA-DRB1*03). HLA-DQB1*05:02 and HLA-DRB1*15:01 are more frequent in NMOSD [5]. Even though we entertained the possibility of genetic factors at play for our described NMOSD cases, we could not do genetic testing for them because of limited affordable capacity for this in Uganda. Overall, although the genetic mechanisms for familial NMOSD are unascertained, it has been suggested that cytokine and cytokine receptor coding genes may have a significant role [6]. Specific factors incline individuals towards AQP4 specific autoimmunity [1].

In conclusion, we add to existing literature and hypothesize that NMOSD appears to show a complex genetic background. To our knowledge, this is the first report in Sub-Saharan Africa, of familial AQP4-IgG positive NMOSD presenting with clinical heterogeneity between first degree relatives. A better understanding of the pathogenic mechanisms involved, including genome wide studies for particular risk loci for familial NMOSD, will be pivotal for future preventative and therapeutic strategies.

## Data Availability

The data used and/or analyzed during the current study are available from the corresponding author on reasonable request.

## Abbreviations

AQP4-IgG: Aquaporin-4 Immunoglobulin
NMOSD: Neuromyelitis Optica Spectrum Disorder
HAART: Highly Active Antiretroviral Therapy
FLAIR: Fluid Attenuated Inversion Recovery
MRI: Magnetic Resonance Imaging
CSF: Cerebrospinal Fluid
HSV: Herpes Simplex Virus
VZV: Varicella Zoster Virus
HHV6: Human Herpesvirus-6
PCR: Polymerase Chain Reaction
EBV: Epstein, Barr virus
VL: Viral Load
TB: Tuberculosis
FTA-ABS: Fluorescent Treponemal Antibody Absorption
CRP: C-reactive protein
dsDNA: Double Stranded Deoxyribonucleic Acid
COVID: Corona Virus Disease
HLA: Human Leukocyte Antigen
